# Peripheral Cornea Crosslinking Before Deep Anterior Lamellar Keratoplasty

**Published:** 2020-03-27

**Authors:** Mohammed Ziaei, Akilesh Gokul, Hans Vellara, Dipika Patel, Charles NJ McGhee

**Affiliations:** 1 Department of Ophthalmology, New Zealand National Eye Centre, Faculty of Medical & Health Sciences, University of Auckland, New Zealand

**Keywords:** Cornea, Crosslinking, Keratoconus, Ectasia

## Abstract

Since Cornea crosslinking (CXL) has been proven to halt progression and biomechanically stabilize keratoconus, we hypothesized that CXL of the corneal periphery 3 months prior to corneal transplantation can reduce the incidence of recurrent ectasia by strengthening the peripheral corneal tissue and causing apoptosis of diseased peripheral host keratocytes. Thus, the aim of this case-report was to propose a novel peripheral CXL technique prior to keratoplasty and evaluate its safety. A 22-year-old woman was admitted with advanced right keratoconus and corrected distance visual acuities of 20/30 in the right eye and 20/200 in the left eye with a manifest refraction of -3.00D/ -8.00D × 36° and -17.00D/ -11.50D × 90°, respectively. The proposed treatment involved crosslinking of peripheral corneal tissue (6.5-9.5mm), sparing the central cornea and limbus, three months prior to corneal transplantation as a means of biomechanically strengthening the peripheral cornea tissue. This procedure was feasible and safe with repopulation of the peripheral cornea with keratocytes, no significant endothelial cell loss and a routine postoperative course following CXL and DALK. This method might reduce or eliminate the need for repeat corneal transplantation in patients with recurrent ectasia. Further studies are needed to confirm the results.

## INTRODUCTION

Keratoectasia is a progressive thinning disorder of the cornea, which compromises the stromal collagen matrix, leading to protrusion as well as alteration of corneal shape and often significant degrees of myopia and astigmatism [1]. Keratoconus is the most common form of keratoectasia with an incidence of 1.3 to 25 per 100,000 and a reported prevalence of 50 to 230 cases per 100,000 [2]. There is a growing body of evidence that implicates genetic factors in the pathogenesis of keratoconus. In keratoconus, total corneal collagen content is normal but changes in stromal collagen fiber and extracellular matrix components, keratocyte morphology and cell-matrix interactions have been reported. The above changes lead to lamellar slippage and subsequent reduction in corneal stiffness, leading to corneal apex protrusion and increased corneal power [3]. 

Conservative management of keratoectasia typically involves the use of spectacle correction in early disease. Contact lens correction in the form of rigid gas permeable contact lenses are typically required in more advanced disease and is the treatment of choice for most patients with keratoconus. 

However, surgical intervention is indicated in patients with advanced disease in whom contact lenses are not able to provide sufficient visual rehabilitation or if intolerant to contact lenses. For advanced cases of ectasia, corneal transplantation preferably with deep anterior lamellar keratoplasty (DALK), or alternatively penetrating keratoplasty (PK) remains the treatment of choice [4, 5]. 

Cornea crosslinking (CXL) is a relatively new treatment of keratoectasia which aims to stabilize the ectatic process using a combination of ultraviolet-A light and a chromophore (Riboflavin). Whilst CXL has been shown to reduce the need for the more invasive keratoplasty procedure, corneal transplantation is still required in a significant proportion of patients with keratoconus, with large studies reporting a keratoplasty rate of 11.8% after 8 years and 18.8% over 20 years [6].

There are multiple reports of recurrent ectasia, sporadic recurrence of ectatic process in recipients of a corneal transplant. Recurrent ectasia has been estimated to occur in 6-11% of transplants up to two decades or more after surgery, based on clinical and histopathological evaluation [7]. The mean reported latency of recurrence in these patients is approximately 19 years, which is analogous to the natural progression of keratoconus [8]. Ultimately, the associated thinning and bowing of the peripheral cornea leads to significant corneal distortion and uncorrectable visual impairment, requiring repeat transplantation for visual rehabilitation.

Recurrent ectasia is frequently bilateral and can recur after repeat transplantation. Host factors, rather than donor ones, are more responsible for the disorder. A slow migration of abnormal host keratocytes into the donor corneal button has been suggested as a possible pathological mechanism, which has been histologically demonstrated in patients undergoing repeat PK [9]. 

Since CXL has been proven to halt progression and biomechanically stabilize keratoconus [10, 11], we hypothesized that CXL of the corneal periphery 3 months prior to corneal transplantation can reduce the incidence of recurrent ectasia by strengthening the peripheral corneal tissue and causing apoptosis of diseased peripheral host keratocytes. The intervention possibly prevents complete repopulation of the graft host junction with host keratocytes and reduces the risk of recurrent ectasia. This study aimed to evaluate the safety of such a procedure in a patient undergoing keratoplasty. 

## METHODS

Surgical Technique

**Figure 1:**
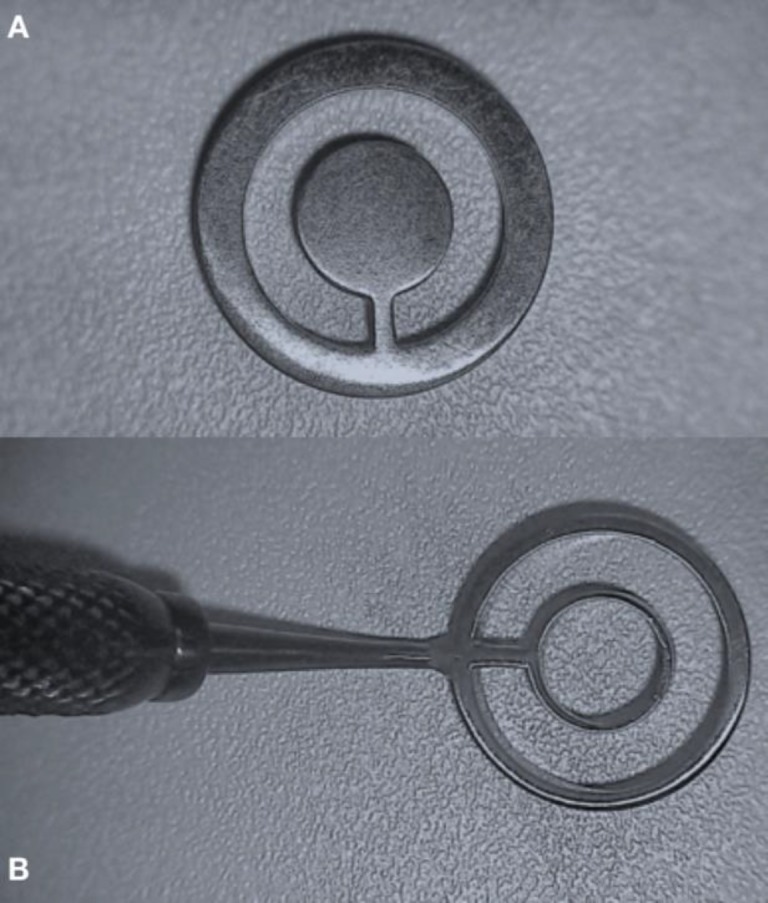
A. Custom Designed Corneal Well Facilitating Peripheral Corneal Epithelial Debridement and Shielding of the Corneal Limbus. B. Custom Designed Corneal Shield, Protecting the Central Cornea and Limbus From Ultraviolet A (UV-A) Irradiation

The surgical procedure was conducted under aseptic conditions. Topical anesthetic drops administered and a speculum placed between the eyelids. Using an operating microscope and a custom designed ophthalmic well (figure 1A), 20% ethanol solution was applied to the peripheral 6.5-9.0 mm corneal zone over 20 seconds and the epithelium was thereafter removed using a blunt spatula. 

The CXL procedure started with application of riboflavin solution composed of dextran-free riboflavin 0.1% with hydroxyl, propyl, methyl and cellulose (Collagex Rapid, LightMed, San Clemente, Calif., USA), with 10 minutes of corneal soaking. Collagen crosslinking was performed at a diameter of 11mm using the LightLink CXL UV-A source (LightMed, San Clemente, Calif., USA) system with 3 minutes of continuous UV-A exposure at 30 mW/cm2 and a total energy dose of 5.4 J/cm2.

**Figure 2:**
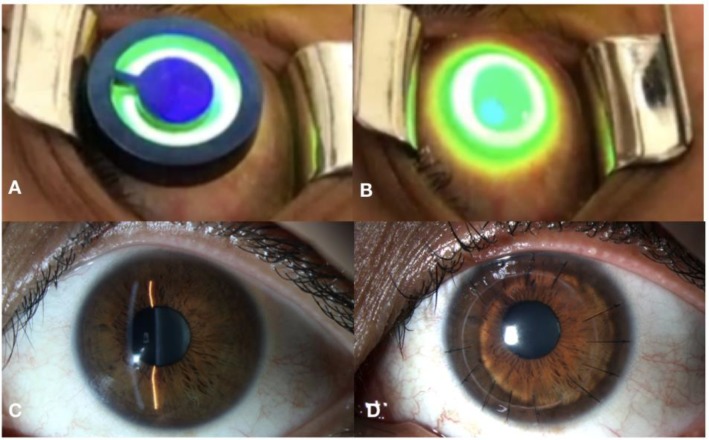
A. Intraoperative Image of the Peripheral Cornea Crosslinking (CXL) Procedure With the Corneal Shield. B. Intraoperative Image of the Peripheral CXL Procedure Without the Corneal Shield. C. Postoperative Image of the Right Eye 3 Months Following Peripheral CXL. D. Postoperative Image of the Right Eye 12 Months After DALK and 15 Months After Peripheral CXL

During UV-A irradiation, the central cornea and corneal limbus were protected using a custom designed corneal shield (figure 1 B), to avoid central corneal endothelial and limbal stem cell damage (figure 2A & 2B). Treated eyes were dressed by a silicone–hydrogel soft contact lens (PureVision; Bausch+Lomb Inc., Rochester, NY) for 3 days. Ciprofloxacin 0.3% (Alcon Laboratories Inc., Fort Worth, TX) and fluorometholone 0.1% (Santen Pharmaceutical Co, Ltd) eye drops 4 times a day were administered for one week and one month, respectively. 

## RESULTS

**Case report**

**Figure 3:**
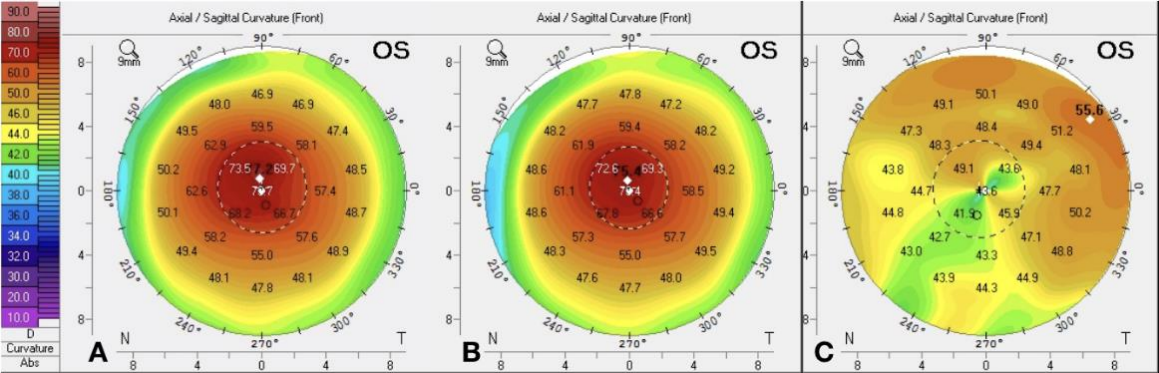
A. Tomography Scans Prior to Peripheral Cornea Crosslinking (CXL). B. Tomography Scan 3 Months Following Peripheral CXL. C. Tomography Scan 12 Months Following Deep Anterior Lamellar Keratoplasty (DALK).

**Figure 4:**
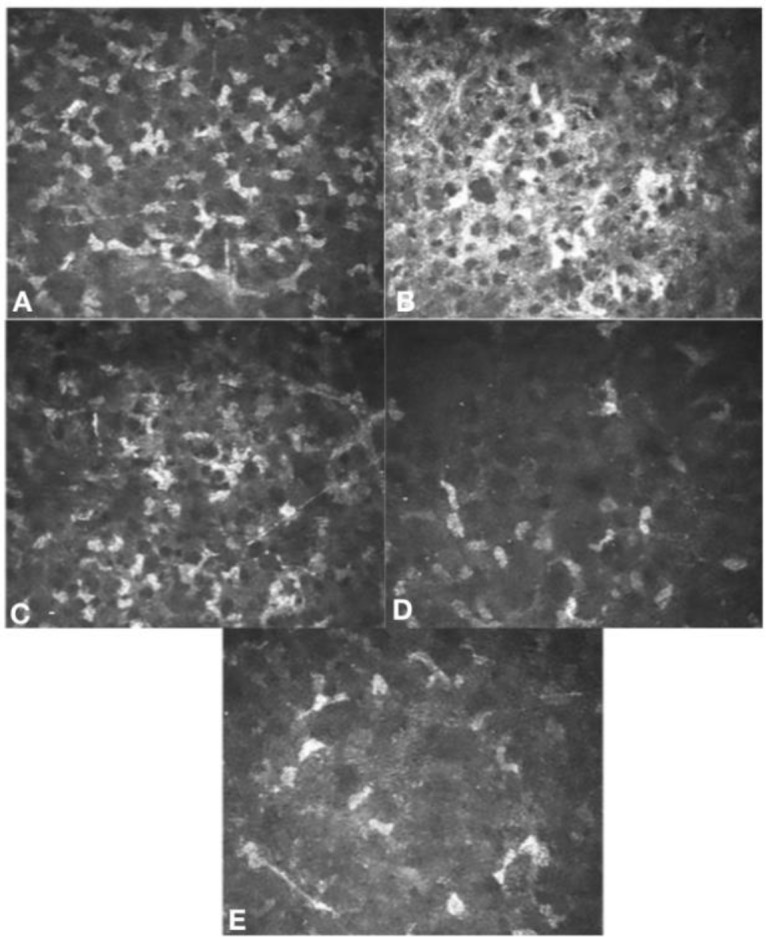
Laser scanning in vivo confocal microscopy (Heidelberg Retina Tomograph III Rostock Corneal Module (HRTIII) (Heidelberg Engineering, GmBH, Germany)) images of superior anterior corneal stroma, 7mm from the corneal apex. A. Prior to peripheral crosslinking, demonstrating the typical keratocyte appearance and density in a patient with keratoconus.B. One-month post-peripheral crosslinking, demonstrating the expected appearance of a reduction in keratocyte density with hyper-reflective cytoplasm of remaining keratocytes and extracellular lacunae. C. Three months post-peripheral crosslinking, keratocyte repopulation and improvement in hyper-reflectivity of cytoplasm and extracellular lacunae. D. Three months post- Deep Anterior Lamellar Keratoplasty (DALK), early repopulation of keratocytes. E. Twelve months post-DALK, further repopulation of keratocytes

**Figure 5:**
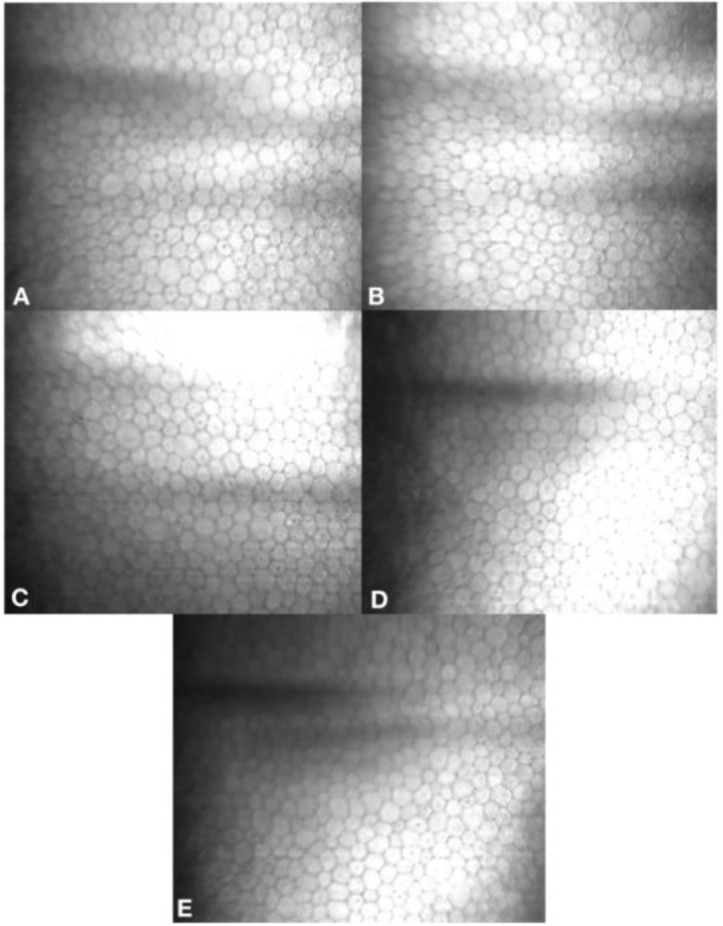
Slit scanning in vivo confocal microscopy Confoscan 4 (Nidek Technologies, Gamagori, Japan) images of superior corneal endothelium, 7mm from the corneal apex. A. Prior to peripheral crosslinking, mean cell density was 3073 cells/mm2. B. One-month post-peripheral crosslinking, mean cell density was 3028 cells/mm2. C. Three months post-peripheral crosslinking, mean cell density was 3039 cells/mm2. D. Three months post-DALK, mean cell density was 3139 cells/mm2. E. Twelve months post- Deep Anterior Lamellar Keratoplasty (DALK), mean cell density was 3149 cells/mm2

A 22-year-old woman was referred with advanced right keratoconus and corrected distance visual acuities of 20/30 in the right eye and 20/200 in the left eye with a manifest refraction of -3.00D/ -8.00D × 36° and -17.00D/ -11.50D × 90°, respectively. Slit lamp examination showed mild cornea apical scarring but no signs of previous hydrops. Corneal tomography (Pentacam, Oculus, Germany) revealed advanced ectasia in the left eye with a thinnest pachymetry measurement of 425 μm (figure 3A). Laser scanning *in vivo *confocal microscopy (Heidelberg Retina Tomograph III Rostock Corneal Module (HRTIII) (Heidelberg Engineering, GmBH, Germany)) of the peripheral cornea revealed a normal stromal architecture (figure 4A) and an endothelial cell count of 3073 cell/mm^2^ (figure 5A). The patient did not have any other ocular or medical history, but reported excessive eye rubbing. After discussing treatment options, risks, benefits and alternatives, the patient consented to undergo peripheral CXL followed by corneal transplantation as she was contact lens intolerant. 

The patient underwent peripheral CXL, three months prior to a planned keratoplasty procedure as described above. No postoperative complications were encountered and the cornea fully re-epithelialized by day 3 following peripheral CXL. One month following peripheral CXL, laser scanning *in vivo *confocal microscopy demonstrated a reduction in keratocyte density with hyper-reflective cytoplasm of remaining keratocytes and extracellular lacunae (figure 4 B) as well as a stable endothelial cell count of 3028 cell/mm^2^ (figure 5B). 

Three months following peripheral CXL and prior to undergoing DALK, the patient’s cornea was clear (figure 2C) with no significant change based on corneal tomography (figure 3B). Laser scanning *in vivo *confocal microscopy showed keratocyte repopulation and improvement in hyper-reflectivity of cytoplasm and extracellular lacunae (figure 4 C) as well as an endothelial cell count of 3039 cell/mm^2^ (figure 5C). 

The keratoplasty procedure was performed under general anesthesia, three months following peripheral CXL. A big bubble DALK was completed as described by Anwar [12]. An 8.25 mm full-thickness donor lenticule was then secured over the 8.00mm recipient bed with 16, 10-0 monofilament nylon interrupted sutures. Postoperatively, the patient was given 2-hourly prednisolone acetate 1% (Predforte, Allergan, New Zealand) and 4-hourly chloramphenicol 0.5% eye drops (Chlorsig, Aspen Pharma Pty Ltd, Australia). The postoperative period was uneventful, with *in vivo* confocal scans performed at 3 months post-DALK confirmed adequate repopulation of the graft host junction with keratocytes (figure 4 D) and a normal endothelial cell count of 3139 cell/mm^2 ^(figure 5D). At 12 months’ postoperative, the graft was clear (figure 2D). Uncorrected distance visual acuity was 20/40 with a corrected distance visual acuity (CDVA( of 20/25 with a manifest refraction of -1.50D/-2.00D × 50°. Laser in* vivo* confocal scans performed 12 months following DALK confirmed further repopulation of the graft host junction with keratocytes (figure 4E) and an endothelial cell count of 3149 cell/mm^2 ^(figure 5E). Corneal tomography scans 12 months following surgery demonstrated 3.50D keratometric astigmatism (figure 3C).

The study was approved by the local Health and Disability Ethics Committee, a branch of the Ministry of Health in New Zealand (NTX/08/08/070AM02). Written informed consent was obtained from our patient after fully explaining details of the study. The study was performed in accordance with the Declaration of Helsinki.

Method for cell count: Only cells within the clearest 200 x 200 µm (333 x 333 pixels) region of an image were counted. Cell counts used the “L” method, whereby cell borders/cell nuclei crossing the left and inferior borders were counted and those crossing the right and superior borders were omitted.

## DISCUSSION

Our case report demonstrates the feasibility and safety of a peripheral CXL technique prior to DALK in patients with keratoconus. This is a potential new use for the CXL procedure, which has previously been shown to stabilize the underlying ectatic process in keratoconus with the potential to “reverse” the disease as quantified by visual, refractive and topographic parameters [1, 13]. 

The exact molecular mechanism of crosslinking has yet to be fully elucidated, but experimental data suggests that it occurs via two mechanisms. In the initial phase of crosslinking, the riboflavin molecule is excited to its single or triplet state and photosensitized oxidation of stromal proteins occurs via its interaction with reactive oxygen species in an aerobic environment (Type II mechanism). After oxygen supply is depleted by the crosslinking process, reactive species of radical ions interact with a variety of molecules located in the corneal stroma in an anaerobic environment (type I mechanism). Production of the above molecules in stroma together with photochemical insult provoke wound healing response in the cornea, which results in increased corneal rigidity in the anterior cornea (200um), modulus of elasticity, resistance to enzymatic degradation and stretching, with an associated decrease in swelling percentage and permeability [1]. 

Confocal microscopy studies from early postoperative period up to 36 months after treatment have previously shown changes in the structure and cellularity of the cornea following CXL. Immediately following treatment, keratocyte apoptosis, stromal edema and a significant reduction in anterior stromal nerve fiber density occur. Three to six months following CXL, activated keratocytes gradually repopulate the corneal stroma from the periphery and some studies suggest that new collagen is laid in the stromal matrix [14]. 

Recurrent ectasia was initially thought to occur from incomplete excision of ectatic tissue during initial keratoplasty surgery, but histopathological analysis has shown that the ectatic process recurs due to the migration of abnormal keratocytes from host to donor cornea [15]. The latency period is much shorter in cases of DALK (mean of 2-3 years) compared to PK (average 19 years), proposing that recurrences stem from the underlying pathology in non-excised corneal tissue, which tends to be more in lamellar surgery [15]. Reverse ectasia, whereby a donor cornea with underlying keratoconus has been transplanted in a host requiring transplantation for reasons other than ectasia has also been reported in the literature [16], but this is likely a very rare phenomenon. 

Our proposed peripheral CXL technique aims to crosslink the peripheral corneal rim, which hosts the donor corneal button whilst sparing the typically thin central cornea which is usually the barrier for patients being eligible for conventional CXL, as a minimum de-epithelialized corneal thickness of 400 μm is recommended to avoid potential irradiation damage to the corneal endothelium. However, the corneal periphery is thicker than the corneal centre in normal individuals (612.5±35.3 μm vs. 551.0±39.4 μm) as well as patients with keratoconus (567.6±35.2 μm vs. 492.0±61.7 μm) [17], which allows for safe peripheral CXL, even in those with advanced keratoconus. 

Our modified CXL technique protects the limbal basal epithelium and its associated “niche”, housing corneal stem cells from the direct effect of UV-A rays or free radicals generated during the crosslinking process [18]. Also, a metallic shield over the limbal area protects the limbal epithelial cells from direct exposure to the cytotoxic effects of UV-A radiation. This mechanical shielding will not prevent diffusion of free radicals from the peripherally irradiated cornea tissue. However, diffused free radicals have a short half-life and should not have a clinically significant impact on the homeostatic function of limbal stem cells [18]. We did not observe any clinical evidence of limbal stem cell failure following the procedure. Crosslinking the entire corneal periphery is probably not required as previous studies have demonstrated that whilst the ectatic process is pancorneal, pathological changes seen in the corneal periphery are less pronounced compared to the centre [19]. This is also supported by the observation that the difference between normal and keratoconic corneal thickness profile becomes less pronounced toward the limbus [20]. 

We postulate that peripheral CXL procedure three months prior to keratoplasty allows for sufficient repopulation of host keratocytes to allow for a normal wound healing response following keratoplasty. This period is however not long enough for total repopulation of host cells, which potentially allows for healthy donor keratocytes, which have previously been shown to survive indefinitely following keratoplasty [21] to concurrently repopulate the peripheral cornea and create a more normal homeostatic environment and physiological balance between collagen production and proteolytic breakdown of the extracellular matrix. 

Further studies are required to fully evaluate the rate at which collagen is degraded and resynthesized in the cornea, as this would impact the durability of peripheral CXL treatment. Some investigators have estimated that this process takes 6 to 7 years based on experimental data, but the true turnover process of collagen in the adult human cornea is currently unknown [22]. Other potential benefits of peripheral CXL procedure include refractive advantages as enhancing the biomechanical rigidity of the graft host junction may protect against induction of astigmatism and higher order aberrations following surgery by reducing the effect of forces at this junction [23]. Reduced keratocyte cell density of the host following treatment can also potentially decrease the risk of immunological rejection [24].

Our study had a number of limitations as it was a single case report with a modest follow-up of 12 months. Larger cohorts of patients, a longer follow-up period, *ex-vivo *histopathological as well as *in-vivo *biomechanical evaluation are required to fully understand the effect of peripheral CXL prior to DALK. 

## CONCLUSION

The benefits of developing a successful method of preventing recurrent ectasia are great and this intervention has the potential to significantly reduce the need for repeat keratoplasty. However, a number of challenges remain before peripheral CXL can be adopted routinely. These include further *in vivo* safety and efficacy studies to ensure that following CXL, regenerated peripheral keratocytes are functionally and metabolically stable and capable of hosting a donor graft. More studies should be performed to examine the cost-effectiveness of routine peripheral CXL prior to widespread adoption of this technique. 

## DISCLOSURE

Ethical issues have been completely observed by the authors. All named authors meet the International Committee of Medical Journal Editors (ICMJE) criteria for authorship of this manuscript, take responsibility for the integrity of the work as a whole, and have given final approval for the version to be published. No conflict of interest has been presented. Funding/Support: None. The datasets analyzed during this study are available from the corresponding author on reasonable request. The authors would like to thank and acknowledge the support of Epsilon, USA for their generous help designing and manufacturing the custom made well and shield. 
